# Dr. Edward A. Millring

**Published:** 1904-01

**Authors:** 


Dr. Edward A. Millring, formerly of Buffalo, died at Stockton, N. Y., December 19, 1903, in the thirty-fifth year of his age. He graduated in medicine from Niagara University in 1890, and practised in Buffalo until about two years ago, when he removed to Stockton. He was the youngest son of William S. Millring of this city.

